# Transferrin Functionalized Liposomes Loading Dopamine HCl: Development and Permeability Studies across an *In Vitro* Model of Human Blood–Brain Barrier

**DOI:** 10.3390/nano8030178

**Published:** 2018-03-20

**Authors:** Antonio Lopalco, Annalisa Cutrignelli, Nunzio Denora, Angela Lopedota, Massimo Franco, Valentino Laquintana

**Affiliations:** 1 Department of Pharmacy–Drug Sciences, University of Bari “Aldo Moro”, 4 E. Orabona st, 70125 Bari, Italy; antonio.lopalco@uniba.it (A.L.); nunzio.denora@uniba.it (N.D.); angelaassunta.lopedota@uniba.it (A.L.); massimo.franco@uniba.it (M.F.); valentino.laquintana@uniba.it (V.L.); 2 Institute for Physical and Chemical Processes (IPCF)-CNR, SS Bari, 4 E. Orabona st, 70125 Bari, Italy

**Keywords:** dopamine, liposomes, drug delivery, transferrin, hCMEC/D3 cells, blood brain barrier

## Abstract

The transport of dopamine across the blood brain barrier represents a challenge for the management of Parkinson’s disease. The employment of central nervous system targeted ligands functionalized nanocarriers could be a valid tactic to overcome this obstacle and avoid undesirable side effects. In this work, transferrin functionalized dopamine-loaded liposomes were made by a modified dehydration–rehydration technique from hydrogenated soy phosphatidylcoline, cholesterol and 1,2-stearoyl-*sn*-glycero-3-phosphoethanolamine-*N*-[carboxy(poly(ethylene glycol)-2000)]. The physical features of the prepared liposomes were established with successive determination of their endothelial permeability across an *in vitro* model of the blood-brain barrier, constituted by human cerebral microvascular endothelial cells (hCMEC/D3). Functionalized dopamine-loaded liposomes with encapsulation efficiency more than 35% were made with sizes in a range around 180 nm, polydispersity indices of 0.2, and positive zeta potential values (+7.5 mV). Their stability and drug release kinetics were also evaluated. The apparent permeability (P_e_) values of encapsulated dopamine in functionalized and unfunctionalized liposomes showed that transferrin functionalized nanocarriers could represent appealing non-toxic candidates for brain delivery, thus improving benefits and decreasing complications to patients subjected to L-dopa chronical treatment.

## 1. Introduction

The delivery of active pharmaceutical ingredients to the central nervous system (CNS) represents the most important challenge for the management of the symptoms of Parkinson’s disease (PD) and other neurodegenerative disorders, because of the various defensive barriers surrounding the brain [1,2]. It is well established that many CNS-active molecules, such as dopamine (DA), do not penetrate across the blood–brain barrier (BBB) to enter the CNS, because of their high polarity, ionized state at physiological pH and/or the deficiency of endogenous cellular membrane transporters located within the brain endothelium, which forms the blood vessel walls [3,4,5]. Only small molecules with adequate hydrophilic/lipophilic balance and molecular weight can be absorbed passively into the CNS, if not substrates for the ABC (ATP-binding cassette) transporters efflux pumps. Different methodologies have been developed to raise the delivery of therapeutics for CNS diseases, including the development of CNS-targeted pro-drugs or co-drugs [6,7,8,9] and functionalized nanocarriers with uptake-facilitating ligands [10,11,12].

Until today, the most successful therapy for the management of PD is represented by L-dopa (LD), a bioprecursor of DA, that crosses the BBB through the active transport mechanism for amino acids and, once in the brain, is metabolized and transformed to DA by the enzyme dopa decarboxylase [13,14,15]. Even though LD improves the PD manifestations in the early phases of the disorder, an excessive peripheral LD bioconversion into DA from within the peripheral nervous system produces several unwanted secondary effects. In detail, clinical and preclinical investigations have shown that LD long-term use is associated with anomalous spontaneous movements, psychiatric complications and DA- or LD-induced neurotoxicity [16,17,18].

**Figure 1:**
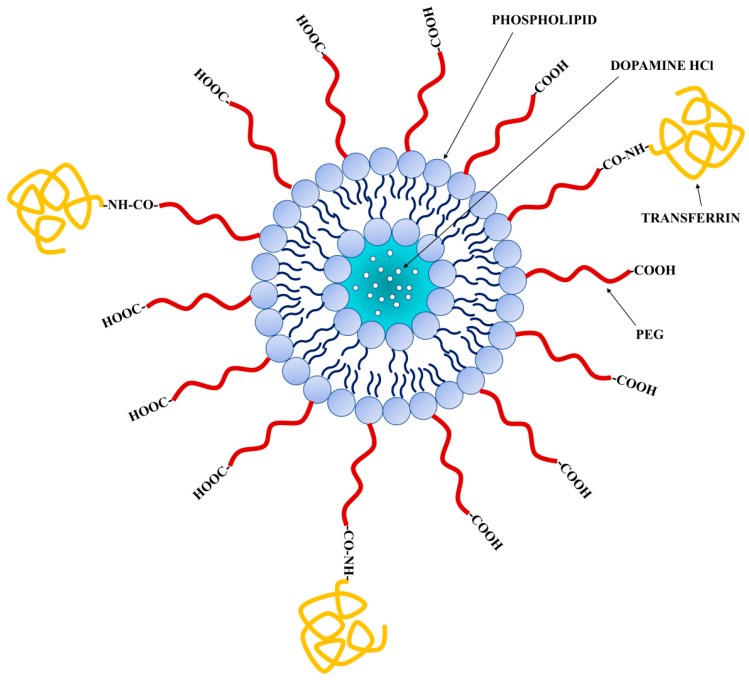
Schematic representation of functionalized DA·HCl-LPs. LPs are made of a phospholipid bilayer, which encloses an aqueous center. The aqueous space incorporates the hydrophilic DA∙HCl. Hydrophilic polymer polyethylene glycol (PEG) coats the ligand-targeted LPs.

In this context, to overcome these disadvantages, a promising strategic delivery system to enhance BBB penetration by DA is the use of nanocarriers such as liposomes (LPs) decorated with uptake-facilitating ligands (Figure 1). In particular, the active targeting approach could be reached using LPs decorated with transferrin (β-1 glycopeptide) (Tf), a hydrophilic carrier that regulates the extracellular iron level in human fluid by binding and sequestering it. In fact, Tf receptor, a dimeric transmembrane glycoprotein, certainly represents a successful target molecule since it as well as being overexpressed in different malignant cells that require high levels of iron for their growth is also localized on the endothelia surface of brain capillaries that comprise the BBB [19,20,21]. Numerous researchers used the targeting to Tf receptor for improving the BBB transport of drugs [22,23].

In this study, we have encapsulated the hydrophilic drug dopamine hychloride (DA∙HCl) into Tf functionalized and unfunctionalized LPs (DA·HCl-LPs). These nanocarriers were made by a dehydration-rehydration technique and their particle sizes, polydispersity index, zeta potential and encapsulation efficiency values were determined. Their stability and drug-release behavior were also evaluated. An additional goal in this work was to study the permeability of the functionalized and unfunctionalized DA·HCl-LPs across an *in vitro* model of the BBB, constituted by human cerebral microvascular endothelial cells (hCMEC/D3), using a well-established procedure.

## 2. Materials and Methods

### 2.1. Materials

Dopamine hydrochloride (DA∙HCl, MW = 189.64 g/mol), cholesterol (Chol), Triton X-100, *N*-(3-dimethylamino-propyl)-*N*’-ethylcarbodiimide hydrochloride (EDC), *N*-hydroxysulfosuccinimide (S-NHS) and Tranferrin (Tf) were bought from Sigma-Aldrich (Milan, Italy).

Hydrogenated soy phosphatidylcoline (Phospholipon 90H, PC) was a gift of Natterman Phospholipids GmbH (Koeln, Germany). 1,2-stearoyl-*sn*-glycero-3-phosphoethanolamine-*N*-[carboxy(poly(ethylene glycol)-2000)] (DSPE-PEG_2000_-COOH) was purchased from Avanti Polar Lipids (Alabaster, AL, USA).

For cellular transport studies luciferin yellow was bought from Sigma-Aldrich (Milan, Italy); Transwell^®^ permeable supports were from Corning (Corning, NY, USA). All the media and supplements for cell culture were bought from Life Technologies, Thermo Fisher Scientific (Waltham, MA, USA). Other materials used in this study were of analytical grade.

### 2.2. Quantification of DA∙HCl

High-performance liquid chromatography (HPLC) analysis was used to detect and quantify DA∙HCl. The HPLC station and the column were the same previously described by Lopedota et al. [24] making a change to the mobile phase which in this case was constituted by 90/10 *v*/*v* 0.020 M potassium phosphate buffer (pH 2.8)/acetonitrile mixture. The flow rate was kept at 1.0 mL/min, the eluent was continuously monitored at a wavelength of 280 nm and in these conditions DA∙HCl retention time was about 6.5 min. Calibration curves were obtained solubilizing DA∙HCl in the same mobile phase and were linear over the tested concentration range (from 0.85 mg/mL (4.48 × 10^−3^ M) to 0.0085 mg/mL (4.48 × 10^−5^ M)).

### 2.3. Preparation of Unfunctionalized LPs

Unfunctionalized LPs were made using the dehydration-rehydration method with a slight modification [25]. Briefly, PC/Chol in 7/3 molar ratio were solubilized in a chloroform/methanol (2/1 *v*/*v*) mixture and the solvents were removed by a rotary evaporator at 55 °C until a lipid film was obtained. The film was stored under vacuum for 3 h to guarantee whole elimination of the organic solvents and then rehydrated in the dark with a DA∙HCl solution in phosphate buffer pH = 4.5. To avoid oxidation of DA∙HCl all subsequent manipulations of the liposomal suspension were carried out in the absence of light. The resulting LPs were sized by sonication (Branson Sonifier 150, Danbury, CT, USA) alternating three cycles of 60 s each with three cooling cycles of 60 s in an ice bath. The liposomal suspension was freeze-dried for 24 h and then subjected to a controlled rehydration process with demineralized water. The un-loaded drug was removed by ultracentrifugation at 45,000 rpm for 50 min at 4 °C (Beckman L7-55, Life Science, Boston, MA, USA) and the obtained pellet was suspended in phosphate buffer pH = 4.5. Finally, dimension, zeta potential and encapsulation efficiency of the obtained vesicles were determined.

### 2.4. Preparation of Tf Functionalized LPs

The preparation of Tf functionalized LPs was conducted using the procedure described by Paszko et al. [26]. In detail, the initial composition of PC/Chol 7/3 molar ratio was integrated with the 2.5 mol % of DSPE-PEG_2000_-COOH and LPs were prepared following the procedure described in the previous paragraph.

Then, LPs suspension was incubated for 10 min at room temperature with S-NHS and EDC, both dissolved in PBS pH = 4.5. Finally, 120 mg of Tf per mmol of lipid were added and incubated for 12 h at 4 °C to allow the formation of an amide bond between the carboxyl and amine groups of PEGylated lipids and Tf, respectively. The unbound Tf was separated from functionalized vesicles by ultracentrifugation at 50,000 rpm for 2 h, at 4 °C (Beckman L7-55, Life Science, Boston, MA, USA). The recovered pellet containing LPs was suspended in PBS pH = 4.5 and stored in the dark until further manipulations.

In order to investigate the density of Tf on the LPs surface a BCA assay kit was used, evaluating the percentage of Tf exposed on external LPs surface compared to the total amount of Tf used for the conjugation. The absorbance at 595 nm was recorded (PerkinElmer 2030 multilabel reader Victor TM X3, Waltham, MA, USA) and the protein concentration was determined by comparison to a standard curve (0.5 to 30 μg/mL).

### 2.5. Physicochemical Characterization of LPs

For the determination of vesicles dimension and polydispersity index (P.I.) a Zetasizer Nano ZS (Malvern Instrument Ltd., Worcestershire, UK) was used and suspensions were appropriately diluted with demineralized water. The zeta potentials were investigated by laser Doppler velocimetry using the same instrument and diluting all samples with a 1 mM KCl solution to keep the ionic strength constant [27].

Experiments were performed in triplicate and the results were reported with the corresponding standard deviation.

### 2.6. Quantification of DA∙HCl into LPs

The quantity of DA∙HCl encapsulated in liposomal vesicles was expressed as the difference between the total quantity solubilized in the LPs medium and the quantity of non-encapsulated DA∙HCl recovered in the aqueous suspending medium after centrifugation at 45,000 rpm for 50 min at 4 °C (Beckman L7-55, Life Science, Boston, MA, USA). DA∙HCl content was determined by HPLC using the calibration curve obtained as explained in Section 2.2. Results are expressed as encapsulation efficiency (EE) determined as actual drug loading/theoretical drug loading × 100 [28]. Experiments were performed in triplicate.

### 2.7. Freeze-Fracture Electron Microscopy

A sample of DA∙HCl-LPs was examined by transmission electron microscopy after freeze-fracture in the presence of 20% of glycerol as cryoprotectant. In detail, a drop of liposome dispersion, deposited in a small gold pan, was quickly frozen in liquid nitrogen. A freeze-replica apparatus at −100 °C (FR-7000A, Hitachi Science Co., Tokyo, Japan) was used to fracturing the sample and replica was realized by platinum-carbon shadowing and examined with a JEM-1200EX (Japan Electron Co., Tokyo, Japan) transmission electron microscope.

### 2.8. In Vitro Release Studies

1 mL of Tf functionalized and unfunctionalized LPs suspension containing DA∙HCl was put into dialysis sacs (cut-off 3000 MW) and dialyzed against 50 mL of phosphate buffer pH = 4.5 supplemented with α-tocopherol 0.005 M to avoid DA∙HCl oxidation in the release medium. The dialysis was conducted at 37 °C in a shaker bath, 100 μL of external medium were removed at predetermined times interval and analyzed by HPLC for DA∙HCl content, and 100 μL of phosphate buffer were added in order to preserve the sink condition. The experiment was conducted on both functionalized and unfunctionalized LPs for at least three times.

### 2.9. Stability Studies

LPs stability was evaluated by measuring size and polydispersity index by means of light scattering for one month, after appropriate dilution with demineralized water.

### 2.10. Culture of hCMEC/D3 Cells and Endothelial Permeability Experiments

The *in vitro* model of the BBB, constituted by human cerebral microvascular endothelial cell line hCMEC/D3 was obtained from Dr. PO Couraud, Inserm, Paris, France. Culture of these cells was realized as reported by Lopalco et al. in a previous study. [2]. Briefly, cells at passage numbers between 25 and 30 were cultivated onto polyester Transwell^®^ inserts and grown in supplemented media. Cell barrier integrity was verified prior to perform endothelial permeability experiments by means of trans-endothelial electrical resistance (TEER) using an EndOhm meter. Monolayers of human cerebral microvascular endothelial cells with TEER values between 65 and 89 Ohm·cm^2^ were used in this study. The transport of Tf functionalized and unfunctionalized DA-LPs was examined at a concentration of 50 µg/mL of DA∙HCl in LPs. The endothelial permeability of the nano-systems was performed as reported by Lopalco et al. [2]. The quantity of DA∙HCl that had passed through the lipid membrane, constituted by the cell monolayer, was determined using HPLC. In order to determine the apparent permeability values across blank Transwell^®^ inserts, experiments were performed in triplicate without seeding cells in the inserts.

Luciferin yellow transport studies were performed in the same manner explained earlier, except that the sample volumes were 200 μL. The cumulative quantity of luciferin yellow transported was measured by determining the fluorescence of the samples in phenol red-free DMEM at *λ*_ex_ = 480 nm and *λ*_em_ = 530 nm using an FLX800 microplate reader (BioTek Instruments, Inc., Winooski, VT, USA) [29]. A Gen5™ software (BioTek Instruments, Inc., Winooski, VT, USA) was used for the acquisition of the data. The relative quantity of luciferin yellow per unit of volume of solution in the basal chamber was then determined from calibration standards made by serial dilution of the luciferin yellow.

### 2.11. Statistical Analysis

Statistical evaluation of data has been made using GraphPad Prism version 5.0 (San Diego, CA, USA) and statistical significance (*p* < 0.05) determined using a one-way analysis of variance (ANOVA) followed by the Bonferroni post hoc tests.

## 3. Results and Discussion

### 3.1. LPs Characterization

LPs containing DA∙HCl and functionalized with Tf were prepared, as described, using a modification of the Kirby and Gregoriadis procedure since this method is well known to improve entrapment of water soluble drugs [30]. Tf was conjugated to the carboxyl group of PEG on the LPs PC/Chol/DSPE-PEG-COOH surface to obtain PC/Chol/DSPE-PEG-CO-Tf according to the procedure described in the Section 2.4. Then, the fully characterization in terms of dimension, polydispersity index, zeta potential, drug loading and Tf coupling efficiency was carried out. Results are summarized in Table 1.

As can be seen, there is a difference between Tf functionalized and unfunctionalized LPs in terms of size and EE%. In particular, unfunctionalized LPs exhibit a mean diameter of 162.4 ± 3.2 nm and a EE% of 41.5 ± 2.9% while for Tf functionalized LPs we found a value of mean diameter equal to 181.7 ± 7.8 and a EE% of 35.4 ± 1.8%. This behavior is quite in agreement with data found in literature [20,31], the coupling of Tf or other ligands on the surface of liposomal vesicles leads to a slight increase in size, although the values are not different from the statistical point of view (*p* > 0.05). In all cases the PDI was equal to 0.2 and this value indicates the existence of a very uniform liposomal population in terms of dimensional distribution.

The charge on the LPs was found to be positive and small for the two formulations (values in a range from +4.8 to +7.5 mV), with a slight increase for Tf functionalized LPs. This behavior could be ascribed to the existence of positive charged functional groups of Tf. Regarding the coupling efficiency of Tf, it was found to be equal to 48.8 ± 2.6%, compared to the total amount of Tf used for the conjugation.

In order to determine the stability of the obtained liposomal preparations, their size and PDI were evaluated one a week for 1 month, keeping them at 4 °C. Results are shown in Table 2.

It is evident that no significant variations in terms of size and PDI are highlighted, so it is possible to state that vesicles are stable and can be used for next studies.

Moreover, after one month we determined by HPLC the DA∙HCl amount in LPs after vesicles disruption with 0.1% Triton X-100 and filtration with 0.22 μm cellulose acetate membrane filter (Millipore^®^, Milan, Italy). It was found equal to 98.2% of the initial amount with no significant loss due to drug oxidation.

**Figure 2:**
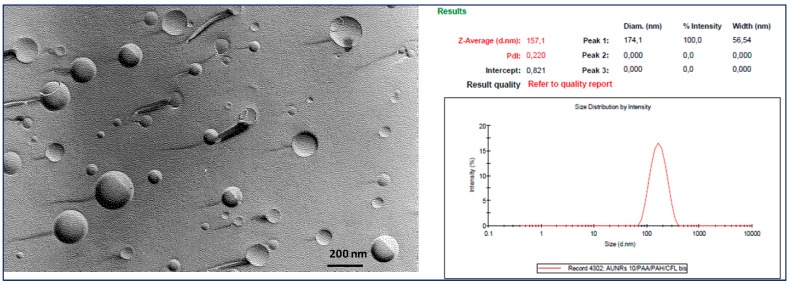
Freeze fracture electron micrograph and size distribution of unfunctionalized DA∙HCl-LPs.

Figure 2 shows the freeze fracture electron micrograph and the size distribution of unfunctionalized DA∙HCl-LPs. Freeze fracture electron microscopy is a powerful technique in the characterization of nanosystems such as micelles, quantum dots, unilamellar and multilamellar liposomes, niosomes and drug crystals because it allows to distinguish between bilayer and non- bilayer structure [32]. Moreover, freeze fracture electron microscopy remains a key tool for investigation of bilayer organization, since it allows to determine the multilamellarity of liposomal systems [32]. As can be seen by micrograph, unfunctionalized DA∙HCl-LPs appeared as SUV (small unilamellar vesicles), as expected having used sonication to homogenize the size distribution, with no ripples on the surface and a fairly uniform distribution in terms of size, according to what has been seen through DLS analysis.

### 3.2. In Vitro Release Studies

**Figure 3:**
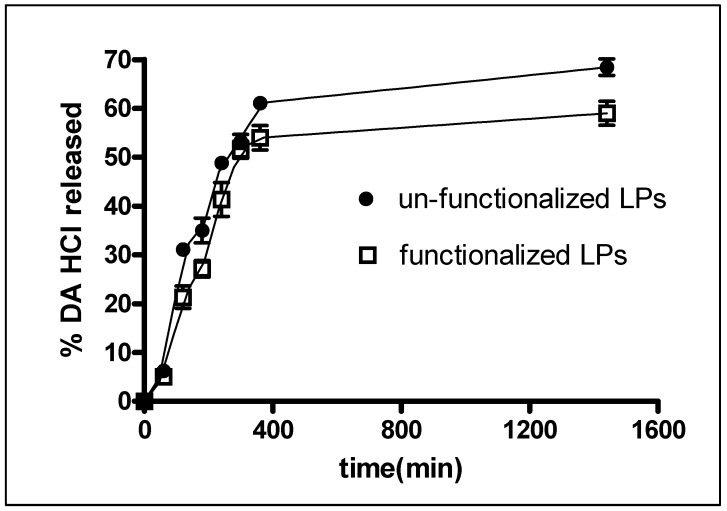
Release profiles of DA∙HCl from LPs. Data are the mean of three determination.

*In vitro* release studies were carried out by dialysis and the obtained cumulative release profiles are reported in Figure 3. The percentage of DA∙HCl released was found to be 59.0 ± 4.2% and 68.4 ± 2.9% for Tf functionalized and unfunctionalized LPs, respectively, after a period of 24 h, without any burst effect. The lower value found for functionalized LPs can be explained by the presence of Tf bound on the LPs surface which results in a decrease in the liposomal membrane permeability, slowing down drug release. This behavior is perfectly in line with what has been reported in the literature [20,22].

### 3.3. In Vitro Transport Analysis

**Figure 4:**
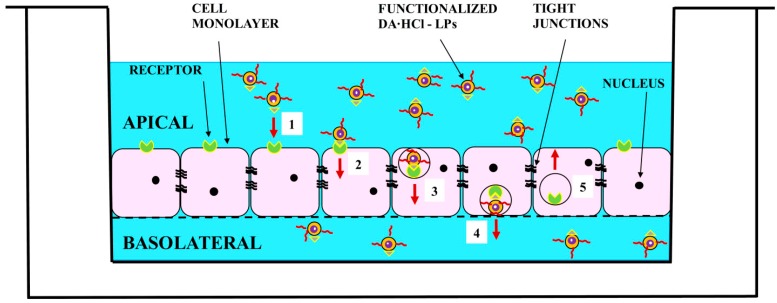
Representation of the endogenous Tf receptor-mediated transcytosis of DA·HCl loaded Tf functionalized LPs across an *in vitro* model of human BBB composed of hCMEC/D3 cell monolayers.

*In vitro* transport of formulations across the BBB was investigated using human hCMEC/D3 cell monolayers. The paracellular permeability (P_e_) of luciferin yellow was evaluated to exclude alterations of the tight junction properties triggered by LPs. In the presence of both functionalized and unfunctionalized LPs the P_e_ value of luciferin yellow was 1.12 ± 0.18 × 10^−3^ cm/min, suggesting no adverse effect on cell monolayer integrity. The data in Table 3 show that the functionalization of LPs with Tf provide a higher permeability across the monolayer compared to unfunctionalized LPs. In detail, the permeability value registered for Tf functionalized DA·HCl-LPs turned out to be equal to 4.97 ± 0.41 × 10^−3^ cm/min versus 0.92 ± 0.24 × 10^−3^ cm/min found for unfunctionalized DA·HCl-LPs, with an increase of about 5 fold. The presence of Tf on the surface of LPs allows vesicles to exploit a mechanism of receptor-mediated endocytosis by means of the Tf receptor which is expressed on the endothelium of the cerebral capillaries (Figure 4). Five steps can describe the mechanism proposed in Figure 4. Initially, LPs decorated with transferrin bind specifically to endothelial receptor (1), resulting in their uptake or endocytosis (2). Intracellularly, LPs are transported in vesicles, that move through the endothelial cytoplasm in apical to basal direction (3), escaping degradation in lysosomes. When the opposing membrane is reached, the vesicle opens towards the basolateral compartment and releases LPs (4). The vesicle with the receptor moves through the endothelial cytoplasm in basal to apical direction (5) [33,34].

## 4. Conclusions

The transport of DA·HCl across the BBB represents one of the main missions for the management of Parkinson’s disease. The employment of CNS targeted Tf functionalized nanoparticles such as LPs could offer a stratagem to overcome this obstacle. In this work, we have prepared both unfunctionalized and Tf functionalized LPs using a method well known to improve the capturing into the vesicles of hydrophilic drugs. Then, we evaluated their dimension, zeta potential, drug loading, Tf coupling efficiency and drug-release behavior. Finally, the permeability (P_e_) through a cellular model of BBB was studied, highlighting how these vesicles are able to permeate through the cell membrane by exploiting a receptor-mediated endocytosis mechanism. The absence of cytotoxicity and the validated technique of preparation make LPs appealing candidates for brain delivery, thus improving benefits and decreasing complications to patients subjected to LD chronical treatment.

## Figures and Tables

**Table 1:** Particle size ± SD, polydispersity index (PDI), zeta potential, and percent of encapsulation efficiency (EE%) of DA∙HCl-LPs unfunctionalized and functionalized with Tf.

Formulation	Size (nm)	PDI	Zeta Potential (mV)	(EE%)
unfunctionalized DA∙HCl-LPs	162.4 ± 3.2	0.20	+4.8 ± 0.9	41.5 ± 2.9
Tf functionalized DA∙HCl-LPs	181.7 ± 7.8	0.20	+7.5 ± 1.2	35.4 ± 1.8

**Table 2:** Particle size ± SD and PDI vales of DA∙HCl-LPs unfunctionalized and functionalized with Transferrin.

Formulation	Week	1	Week	2	Week	3	Week	4
	Size (nm)	PDI	Size (nm)	PDI	Size (nm)	PDI	Size (nm)	PDI
unfunctionalized DA∙HCl-LPs	168.4 ± 2.4	0.20	165.4 ± 1.8	0.25	159.4 ± 3.5	0.19	160.7 ± 1.2	0.21
Tf functionalized DA∙HCl-LPs	186.5 ± 7.8	0.20	175.7 ± 1.3	0.18	182.4 ± 4.1	0.23	179.4 ± 0.8	0.18

**Table 3:** hCMEC/D3 permeability values (P_e_) of DA·HCl-LPs, functionalized DA·HCl-LPs and luciferin yellow ± standard deviation (SD).

Formulation	P_e_ ± SD (cm/min)
Unfunctionalized DA·HCl-LPs	0.92 ± 0.24 × 10^−3^
Tf Functionalized DA·HCl-LPs	4.97 ± 0.41 × 10^−3^
Luciferin yellow	1.12 ± 0.18 × 10^−3^
